# *Porphyromonas gingivalis* infection promotes mitochondrial dysfunction through Drp1-dependent mitochondrial fission in endothelial cells

**DOI:** 10.1038/s41368-021-00134-4

**Published:** 2021-09-03

**Authors:** Tong Xu, Qin Dong, Yuxiao Luo, Yanqing Liu, Liang Gao, Yaping Pan, Dongmei Zhang

**Affiliations:** 1 Department of Periodontics, School of Stomatology, China Medical University, Shenyang, China; 2 Department of Periodontics and Oral Biology, School and Hospital of Stomatology, China Medical University, Liaoning Provincial Key Laboratory of Oral Disease, Shenyang, China

**Keywords:** Mechanisms of disease, Bacterial pathogenesis

## Abstract

*Porphyromonas gingivalis* (*P. gingivalis*), a key pathogen in periodontitis, has been shown to accelerate the progression of atherosclerosis (AS). However, the definite mechanisms remain elusive. Emerging evidence supports an association between mitochondrial dysfunction and AS. In our study, the impact of *P. gingivalis* on mitochondrial dysfunction and the potential mechanism were investigated. The mitochondrial morphology of EA.hy926 cells infected with *P. gingivalis* was assessed by transmission electron microscopy, mitochondrial staining, and quantitative analysis of the mitochondrial network. Fluorescence staining and flow cytometry analysis were performed to determine mitochondrial reactive oxygen species (mtROS) and mitochondrial membrane potential (MMP) levels. Cellular ATP production was examined by a luminescence assay kit. The expression of key fusion and fission proteins was evaluated by western blot and immunofluorescence. Mdivi-1, a specific Drp1 inhibitor, was used to elucidate the role of Drp1 in mitochondrial dysfunction. Our findings showed that *P*. *gingivalis* infection induced mitochondrial fragmentation, increased the mtROS levels, and decreased the MMP and ATP concentration in vascular endothelial cells. We observed upregulation of Drp1 (Ser616) phosphorylation and translocation of Drp1 to mitochondria. Mdivi-1 blocked the mitochondrial fragmentation and dysfunction induced by *P*. *gingivalis*. Collectively, these results revealed that *P*. *gingivalis* infection promoted mitochondrial fragmentation and dysfunction, which was dependent on Drp1. Mitochondrial dysfunction may represent the mechanism by which *P*. *gingivalis* exacerbates atherosclerotic lesions.

## Introduction

As a well-known periodontal pathogen, *Porphyromonas gingivalis* (*P. gingivalis*) is highly related to periodontal destruction.^1^ *P*. *gingivalis* can invade arterial walls and survive within cardiovascular endothelial cells,^2,3^ and multiple studies have reported the existence of *P*. *gingivalis* in atherosclerotic plaques.^4,5^ Atherosclerosis is the pathological basis of diverse cardiovascular diseases. Over the last few decades, researchers have discovered that atherosclerosis is an inflammatory disease, and chronic infection plays a critical role in disease progression.^6^ Several studies have reported that infectious agents can induce cellular and molecular changes in the inflammatory processes of atherosclerosis,^6–9^ and researchers believe that the inflammatory response to the infectious agent may underlie disease acceleration.

*P. gingivalis* has been shown to accelerate atherosclerotic plaque formation in a murine model based on dietary risk factors, genetic susceptibility, and vascular damage.^7–10^ Furthermore, Xie proved that *P*. *gingivalis* could impair the integrity of the endothelium and inhibit its repairability, which is believed to be of primary importance in the pathogenesis of vascular disease.^11^ Generally, increasing evidence supports that *P*. *gingivalis* infection directly affects the progression of atherosclerosis.

The role of infection in atherosclerosis has attracted the attention of numerous researchers, and various studies have indicated an association between mitochondrial dysfunction and the disease.^12–15^ Mitochondrial redox imbalance is involved in the key events of atherosclerosis, and mitochondrial DNA damage can directly promote atherosclerosis and plaque vulnerability. Both acceleration of atherosclerosis and increased mitochondrial oxidant production were detected in the atherosclerosis model.

The ability of *P*. *gingivalis* to manipulate mitochondrial function is intriguing given the link between *P*. *gingivalis* and atherosclerosis. To date, little is known about the impact of *P*. *gingivalis* on the mitochondria of endothelial cells. As described in the limited literature, increased mitochondrial reactive oxygen species (mtROS) production was observed in endothelial cells infected with *P*. *gingivalis*.^16^ Similarly, Zahlten proved that *P*. *gingivalis* phosphoglycerol dihydroceramides induced apoptosis, but not necrosis, in endothelial cells.^17^ Early apoptotic cells showed exposure of phosphatidylserine on the cell surface, followed by the cleavage of procaspases 3, 6, and 9. The authors reported the increased release of apoptosis-inducing factors and indicated that mitochondria were involved in apoptosis.

The two studies mentioned above indicated that *P*. *gingivalis* may play a role in endothelial mitochondrial dysfunction. In the present study, we identified the possible role of *P*. *gingivalis* in mitochondrial dysfunction and further explored the potential mechanism involved. Here, we uncovered mitochondrial fragmentation and mitochondrial dysfunction in endothelial cells infected with *P*. *gingivalis* by immunofluorescence staining and examination of mtROS and membrane potential levels. Moreover, the induced mitochondrial dysfunction was shown to be dependent on Drp1 assembly and GTPase activity. Our findings provide new insights into the potential for *P*. *gingivalis* to exacerbate atherosclerotic lesions, and chemicals or treatments aimed at recovering mitochondrial function represent a probable strategy for preventing *P*. *gingivalis*-induced atherosclerotic disease.

## Results

### Effect of *P*. *gingivalis* on mitochondrial morphology

**Fig. 1:**
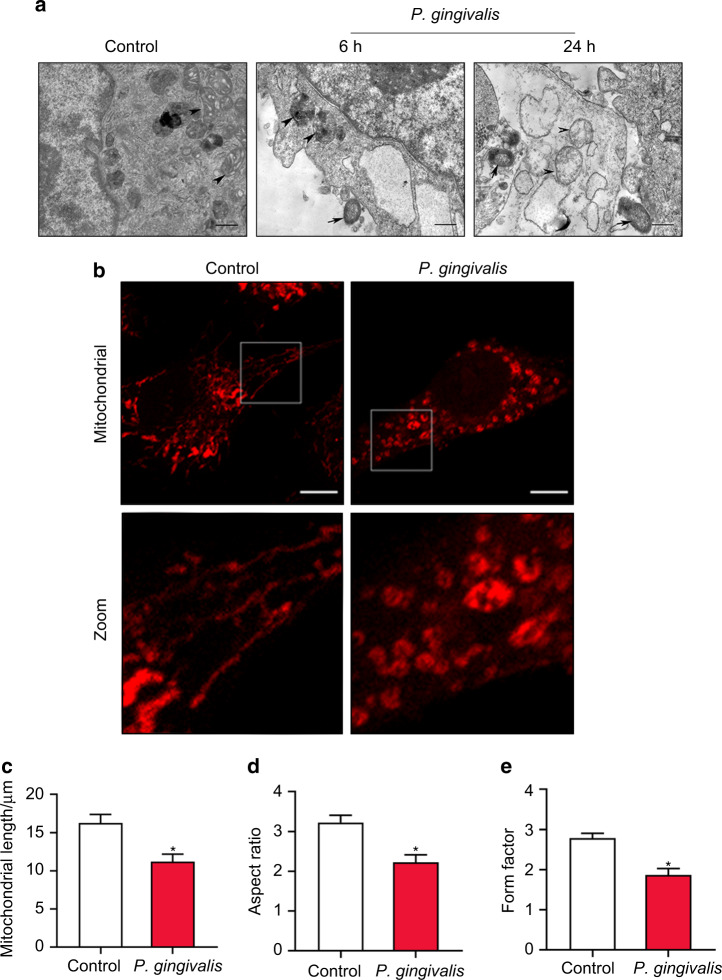
*P. gingivalis* infection triggered excessive mitochondrial fragmentation in endothelial cells. EA.hy926 cells were exposed to *P*. *gingivalis* for the indicated amounts of time (MOI = 100). Cells cultured without *P*. *gingivalis* were used as a control. **a** Mitochondrial morphology was visualized by transmission electron microscopy. Scale bars: 1 µm. Arrow, *P*. *gingivalis*. Arrowhead, mitochondria. **b** Cells were treated with *P*. *gingivalis* for 6 h. Mitochondria were labeled with MitoTracker Red CMXRos staining and observed under confocal laser scanning microscopy. Scale bars: 20 µm. **c** Mitochondrial length was measured by MitoTracker Red. **d** The average aspect ratio (AR, major/minor axis of an ellipse) and **e** form factor (FF, perimeter^2^/4π∙area) of the mitochondria labeled with MitoTracker Red were measured. Smaller mitochondrial length, AR, and FF values represent increased mitochondrial fragmentation. The average length, AR, and FF values were calculated from three images per experiment. The experiments were conducted three times, and the data are presented as the means ± SDs. *P < 0.05 versus the control

Normal mitochondrial morphology is important for the maintenance of mitochondrial function. Here, we examined the changes in the mitochondrial morphology of endothelial cells exposed to *P*. *gingivalis*. As shown in Fig. 1a, the uninfected cells exhibited a long tubular structure with clear internal cristae as determined by electron microscopy. Six hours later, the number of small and punctate mitochondria was increased in the infected cells, accompanied by internal crista cracking and mitochondrial swelling, indicating mitochondrial fragmentation. Twenty-four hours later, most of the mitochondria showed more swelling and puncta, with the internal cristae almost disappearing. Mitochondrial staining showed that normal mitochondria exhibited typical lengths and tubular shapes and formed a highly interconnected network, whereas *P*. *gingivalis* induced mitochondrial network damage. The average mitochondrial length was decreased by 31% at 6 h compared to that of the uninfected cells (Fig. 1b, c). Similarly, the aspect ratio (AR, major/minor axis of an ellipse) and form factor (FF, perimeter^2^/4π∙area) were decreased by 31% and 33%, respectively, after infection (Fig. 1d, e). Our results illustrated that *P*. *gingivalis* led to mitochondrial fragmentation.

### *P*. *gingivalis* induced mitochondrial dysfunction

**Fig. 2:**
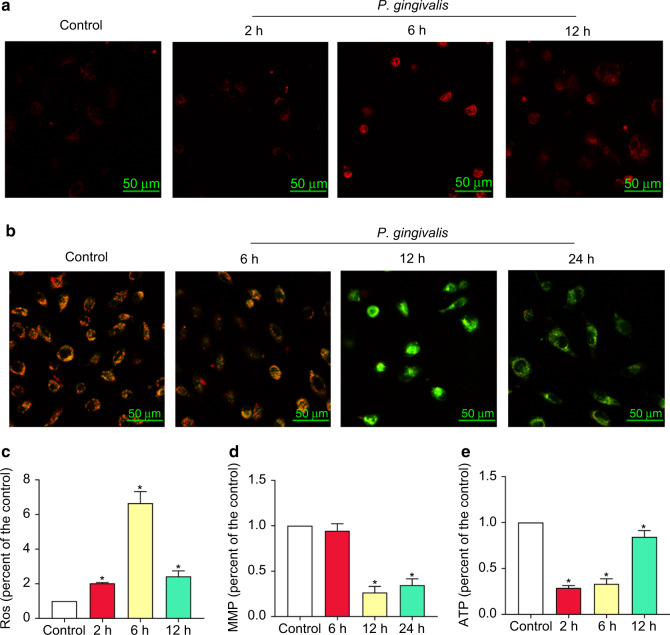
*P. gingivalis* induced mitochondrial dysfunction in endothelial cells. EA.hy926 cells were treated with *P*. *gingivalis* (MOI = 100) for the indicated amounts of time. **a** Mitochondrial ROS (mtROS) levels were determined by MitoSOX Red staining and confocal laser scanning microscopy. Scale bars: 50 µm. **b** The mitochondrial membrane potential (MMP) was measured by JC-1 staining and confocal laser scanning microscopy. Scale bars: 50 µm. **c** Quantification of mtROS levels via flow cytometry. **d** Quantitative of the MMP by flow cytometry. **e** Quantitative analysis of ATP production. The results were obtained from three independent experiments. *P < 0.05 vs the control

We determined the effect of *P*. *gingivalis* on mtROS (Fig. 2a) and mitochondrial membrane potential (MMP, Fig. 2b) by MitoSOX Red staining and JC-1 staining, respectively. The confocal laser scanning microscopy (CLSM) revealed increased mtROS fluorescence intensity in infected cells. These results were further confirmed by flow cytometry analysis. *P*. *gingivalis* induced the accumulation of mtROS as early as 2 h after infection, and the mtROS level was elevated most significantly at the 6 h timepoint (6.57-fold higher than that in the control). Twelve hours later, the mtROS level was obviously decreased but still higher than that of the control (Fig. 2c). Thus, these findings confirmed that *P*. *gingivalis* infection dramatically enhanced the mtROS level. And the influence of *P*. *gingivalis* on the MMP was shown by CLSM. Compared with the control cells, those exposed to *P*. *gingivalis* exhibited a significant shift of red to green fluorescence (Fig. 2b). Qualitative analysis revealed that the MMP levels were decreased by 67% and 63% after 12 and 24 h of infection, respectively. However, the levels were not significantly different from those in the control after 6 h of *P*. *gingivalis* infection (Fig. 2d). These findings suggested that *P*. *gingivalis* decreased the MMP.

Because cellular metabolism maintains energy homeostasis by regulating ATP levels, ATP production was detected to assess the mitochondrial energy metabolism. The ATP content was significantly decreased by 71%, 67%, and 15% at 2, 6, and 12 h after *P*. *gingivalis* infection, respectively, compared with the control group (Fig. 2e).

### Effect of *P*. *gingivalis* on key fusion/fission proteins

**Fig. 3:**
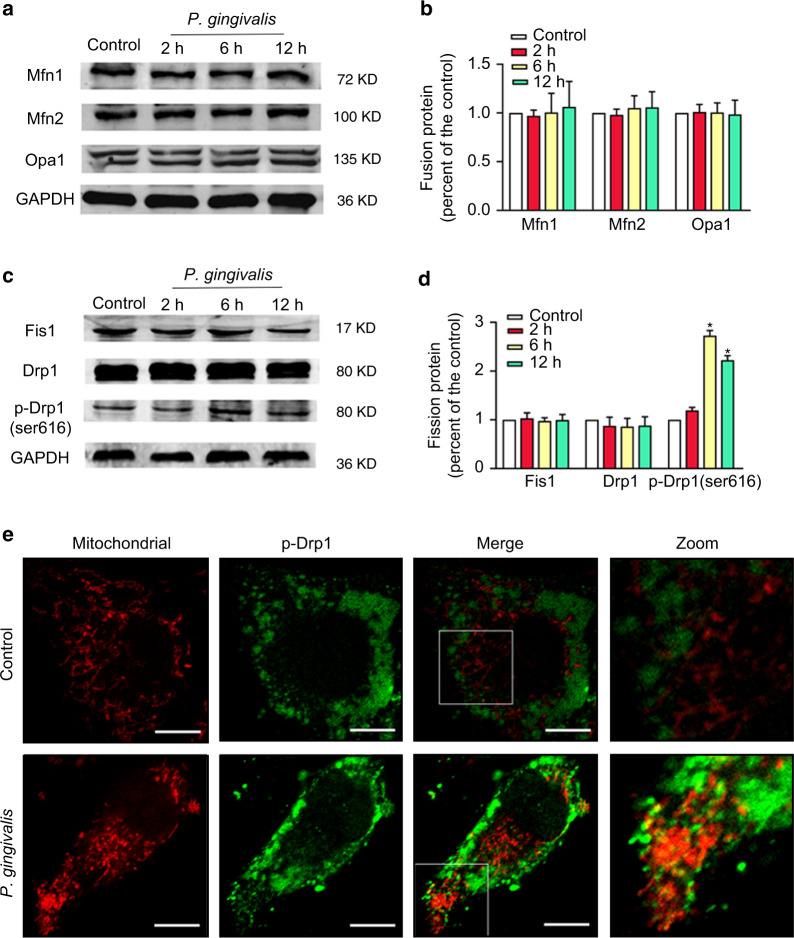
Effects of *P. gingivalis* on key fusion and fission proteins. EA.hy926 cells were infected with *P*. *gingivalis* (MOI = 100) for the indicated amounts of time. Fusion (**a**) and fission (**c**) protein expression was detected by western blot. The relative levels of the proteins compared to the control are shown (**b**, **d**). The fusion protein levels of Mfn1, Mfn2, and Opa1 were not affected by *P*. *gingivalis*. Although the expression levels of the fission proteins (Fis1 and Drp1) were not obviously changed, the p-Drp1 (Ser616) level was significantly increased and peaked at 6 h after *P*. *gingivalis* infection. GAPDH was used as a loading control. The data represent the mean ± SD of three independent experiments. *P < 0.05 vs the control. **e** Colocalization of p-Drp1 (Ser616) and mitochondria in EA.hy926 cells infected with or without *P*. *gingivalis* for 6 h. The mitochondria were stained with MitoTracker Red CMXRos, and p-Drp1 was immunostained with anti-p-Drp1 (green). Then, the cells were observed under a confocal microscope, and representative images were obtained from three independent experiments. Scale bars: 20 µm

Mitochondrial dynamics involve multiple proteins, and the key proteins involved in mitochondrial fusion events are Mfn1/2 and Opa1, whereas Fis1 and Drp1 are involved in mitochondrial fission. Herein, the levels of the mitochondrial fusion proteins Mfn1, Mfn2, and Opa1 were not affected by *P*. *gingivalis* as determined by western blot analysis (Fig. 3a, b). Numerous studies have demonstrated that Drp1 is primarily distributed in the cytosol of normal cells, and phosphorylation of Drp1 at Ser616 promotes its recruitment to the outer mitochondrial membrane in the event of mitochondrial fission. Although the total amounts of Fis1 and Drp1 were not obviously changed, the p-Drp1 (Ser616) level was increased significantly and peaked at 6 h after *P*. *gingivalis* infection. *P*. *gingivalis* increased the expression of p-Drp1 (Ser616) by 2.72-fold compared to that in the control group (Fig. 3c, d). Further immunofluorescence analysis was used to examine the localization of p-Drp1 (Ser616). The signals of p-Drp1 and fragmented mitochondria colocalization were markedly enhanced in the cells exposed to *P*. *gingivalis* for 6 h (Fig. 3e). Taken together, our results indicated that the phosphorylation and recruitment of Drp1 to the outer mitochondrial membrane, rather than the total Drp1 expression, might be the key events leading to mitochondrial dysfunction in *P*. *gingivalis*-infected endothelial cells.

### Effect of Drp1 inhibition on p-Drp1 (Ser616) expression and translocation

**Fig. 4:**
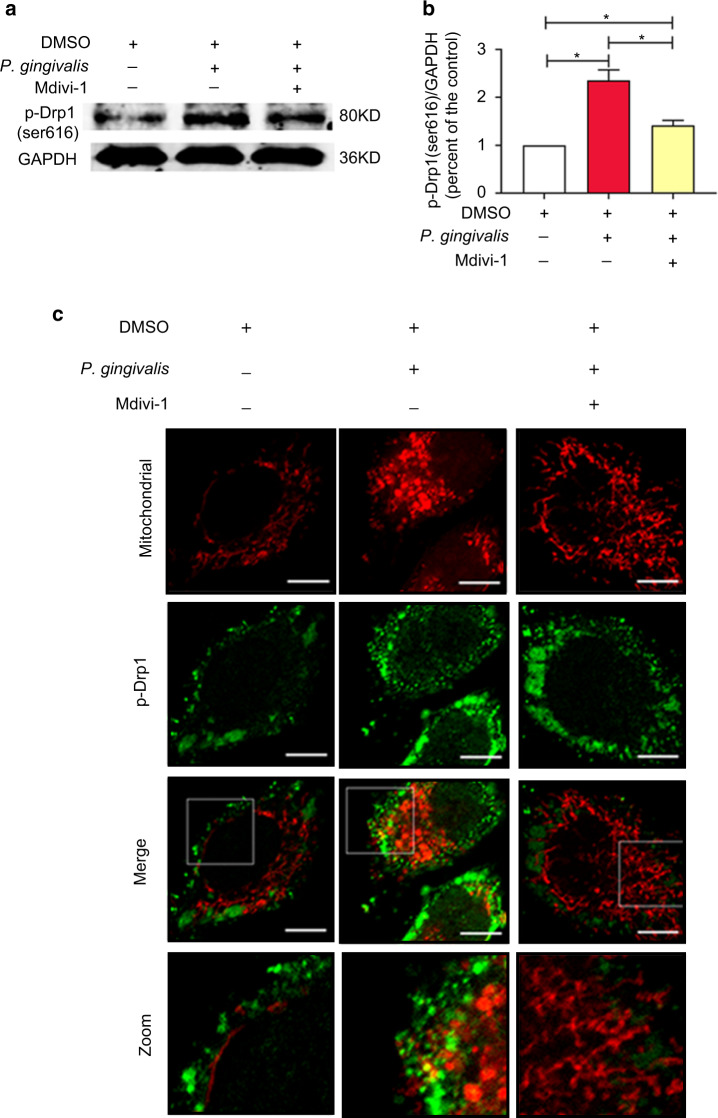
Effect of Drp1 inhibition on p-Drp1 (Ser616) expression and translocation. EA.hy926 cells were pretreated with 20 mM Mdivi-1 or its solvent DMSO for 2 h and then infected with *P*. *gingivalis* for 6 h (MOI = 100). **a** Western blot was used to detect the phosphorylation of Drp1 (Ser616). **b** The relative level of p-Drp1 compared to the control. *P < 0.05. **c** Fluorescence staining of mitochondria (red) and p-Drp1 (Ser616, green) in endothelial cells was performed to detect the effect of Mdivi-1 on p-Drp1 translocation. Three independent experiments were conducted, and the representative images were obtained. Scale bars: 20 µm

Mdivi-1 was added to the cells 2 h before infection to investigate the contribution of Drp1 to mitochondrial dysfunction. *P*. *gingivalis* dramatically increased the level of p-Drp1 (Ser616), which was 2.36-fold higher than that in the uninfected cells. The level of p-Drp1 (Ser616) in the Mdivi-1 group was 1.42-fold higher than that in the control group, and the difference was significantly different (Fig. 4a, b). The cellular distribution of p-Drp1 was also evaluated by immunofluorescence analysis, revealing that *P*. *gingivalis* increased the translocation of p-Drp1 (Ser616) to the mitochondria. The effects of *P*. *gingivalis* were abolished by Mdivi-1 to some extent (Fig. 4c).

### Drp1 mediated *P*. *gingivalis*-induced mitochondrial fragmentation and dysfunction

**Fig. 5:**
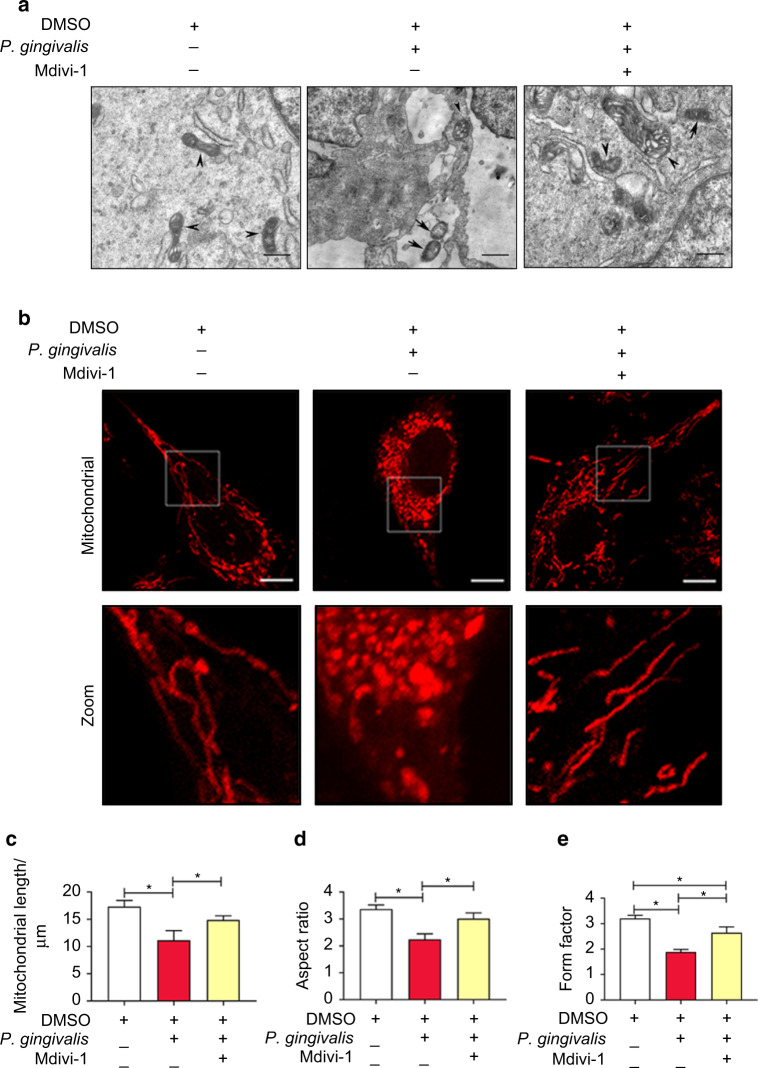
*P. gingivalis*-induced mitochondrial fragmentation was dependent on Drp1. EA.hy926 cells were pretreated with 20 mM Mdivi-1 or DMSO for 2 h and then infected with *P*. *gingivalis* for 6 h. **a** Mitochondrial morphology was visualized by transmission electron microscopy. Scale bars: 1 µm. Arrow, *P*. *gingivalis*. Arrowhead, mitochondria. **b** The mitochondrial network was stained by MitoTracker Red CMXRos and observed under a confocal laser scanning microscope. Scale bars: 20 µm. **c**–**e** Quantitative analysis of mitochondrial morphology was conducted (**c** mitochondrial length. **d** aspect ratio, major/minor axis of an ellipse. **e** form factor, perimeter^2^/4π∙area). The results are presented as the mean ± SD of three independent experiments. *P < 0.05

As mentioned above, most of the mitochondria in the infected cells became punctated and were significantly shortened. However, this effect of *P*. *gingivalis* was significantly suppressed by Mdivi-1 (Fig. 5a). Confocal imaging also indicated that Mdivi-1 efficiently prevented the mitochondrial network loss in endothelial cells exposed to *P*. *gingivalis* (Fig. 5b). Similarly, the mitochondrial length, AR, and FF values were decreased by 36%, 34%, and 41%, respectively, at 6 h after *P*. *gingivalis* infection compared to those in the control. However, Mdivi-1 attenuated mitochondrial fragmentation and improved the reductions in the AR and FF values caused by *P*. *gingivalis*. Compared with those in the infected group, the mitochondrial length, AR, and FF values of the Mdivi-1 group were increased by 1.33, 1.35, and 1.41 fold, respectively. There were no differences in the mitochondrial length and AR values between the control and Mdivi-1 groups (Fig. 5c, e).

**Fig. 6:**
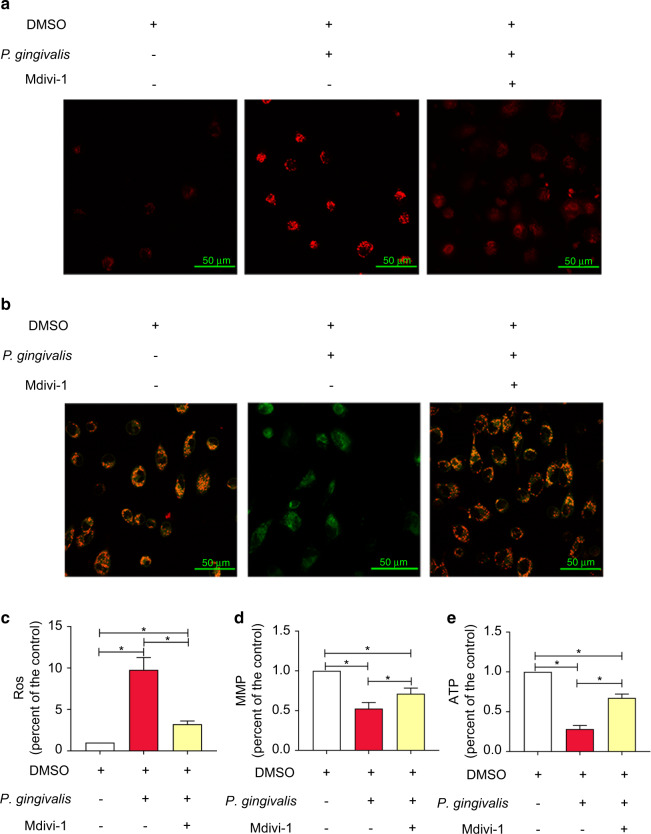
*P. gingivalis*-induced mitochondrial dysfunction was dependent on Drp1 in endothelial cells. EA.hy926 cells were pretreated with 20 mM Mdivi-1 or DMSO for 2 h before being exposed to *P*. *gingivalis* (6 h for mtROS, 12 h for MMP, and 2 h for ATP production) to elucidate the role of Drp1. **a** The mtROS fluorescence intensity was observed under a confocal microscope. Scale bars: 50 µm. **b** The MMP was measured by JC-1 staining and confocal laser scanning microscopy. Scale bars: 50 µm. **c** Quantitative analysis of mtROS levels. **d** Quantitative analysis of the ratio of JC-1 oligomer (red)/monomer (green) fluorescence. **e** Quantitative analysis of ATP production. The data are presented as the mean ± SD of three independent experiments. *P < 0.05

The effect of Drp1 inhibition on mitochondrial ROS and MMP was demonstrated by CLSM in Fig. 6a, b. The flow cytometry analyses showed that *P*. *gingivalis* infection for 6 h increased the mtROS level (9.79-fold higher than that in the control), and this effect was inhibited by pretreatment with Mdivi-1. The mtROS level was significantly reduced by the Drp1 inhibitor (by 67% compared with the *P*. *gingivalis* group) but was still higher than that in the control group. As shown in Fig. 6c, the mtROS level in the Mdivi-1 group was 3.23-fold higher than that in the control. Consistent with the above results, the MMP was decreased significantly after 12 h of infection. However, the CLSM images revealed that Mdivi-1 significantly restored the reduction in MMP caused by infection (Fig. 6b), and quantitative analyses further proved the effect of Mdivi-1. The MMP level was decreased by 53% in the *P*. *gingivalis* group and by 71% in the Mdivi-1 group compared with the control, and the differences among the three groups were significant (Fig. 6d). These results demonstrated that inhibition of Drp1 efficiently rescued the collapse of the MMP.

A similar effect of Mdivi-1 on ATP production was revealed. Compared with that in the control group, ATP production was decreased by 72% after 2 h of infection. However, pretreatment with Mdivi-1 abrogated the ATP depletion (67% of the control), and the inhibition partially restored the mitochondrial dysfunction (Fig. 6e).

## Discussion

Accumulating evidence supports that infection with *P*. *gingivalis*, a key pathogen of periodontitis, is a risk factor for the occurrence and development of atherosclerosis. A proatherogenic response is generated in endothelial cells by the bacterial products released from *P*. *gingivalis* into circulation, and endothelial dysfunction can be induced by the direct invasion of *P*. *gingivalis*.^18^ In addition, *P*. *gingivalis* causes endothelial apoptosis and increases monocyte adhesion to endothelial cells, which are critical events in the development of atherosclerosis.^19^ Our previous research also proved that *P*. *gingivalis* infection upregulates the ICAM-1 expression in endothelial cells, which promotes monocyte-endothelial cell adhesion.^20–22^ In this event, platelet aggregation is triggered, and thrombus formation is initiated by the injured endothelium.^23^ Upon atherosclerosis progression, the infectious *P*. *gingivalis* translocates from endothelial cells to smooth muscle cells.^24^ *P*. *gingivalis* survival in human vascular cells and its transmission from one cell to another promote the inflammatory cycle and contribute to the development of atherosclerosis.

However, complete elucidation of the molecular mechanisms underlying the association of *P*. *gingivalis* with atherosclerosis still requires further research. In the last 20–30 years, researchers have realized that mitochondria are very dynamic based on their continuously changing overall morphology. Both mitochondrial dynamics (including the variability in shape due to fission or fusion) and mitochondrial calcium, which participates in endoplasmic reticulum-mitochondrial coupling, manipulate the integrity and function of mitochondria.^14,25,26^ A renewed appreciation for frequent mitochondrial fission and fusion in the context of cell survival emerged fairly recently.^26^ It is now well accepted that mitochondrial dynamics are highly controlled and crucial for maintaining robust mitochondrial structure and functions, including cellular development, homeostasis, and apoptosis.^27–29^ Defective regulation of these functions causes cellular dysfunction, leading to metabolic and neurological disorders. Numerous experimental studies have shown that mitochondrial dysfunction promotes the onset and progression of atherosclerosis.^15,30–32^ Mitochondrial dysfunction plays a role in atherosclerosis by inducing excessive ROS accumulation, mitochondrial oxidative stress injury, imbalanced mitochondrial dynamics, and an insufficient energy supply. Under pathophysiological conditions, mitochondrial DNA is mutated, thereby reducing the efficiency of oxidative phosphorylation and ATP production, followed by excessive production of ROS and a considerable decrease in the MMP. All these impaired mitochondrial processes were shown to be involved in accelerating the development of atherosclerosis in animal studies.^15,33^

Several studies have reported the influence of *P*. *gingivalis* on mitochondrial function in gingival epithelial cells. Yilmaz,^34^ Boisvert,^35^ and Mao^36^ et al. found that *P*. *gingivalis* could inhibit the chemically induced apoptosis of gingival epithelial cells. *P*. *gingivalis* infection functions at the mitochondrial level, as it blocks MMP depolarization and cytochrome c release or the activation of the effector caspase-3. Yilmaz et al. proved that *P*. *gingivalis* could manipulate mitochondrial functions through the PI3K/Akt pathway, thereby resisting primary gingival epithelial cell clearance by an antiapoptotic effect.^34^ In a later study, the authors found that *P*. *gingivalis* could not only inhibit the production of ROS stimulated by eATP-P_2_X_7_ signaling but also circumvent the eATP/NOX2-ROS-antibacterial pathway to colonize and survive in epithelial cells.^37,38^ However, there are conflicting opinions on the effect of *P*. *gingivalis* on epithelial cell mitochondria, as Li demonstrated that *P*. *gingivalis* induced MMP depolarization and apoptosis in human epithelial (KB) cells. Moreover, *P*. *gingivalis* was shown to upregulate apoptosis-inducing factor (AIF) but did not activate caspase-3 during apoptosis.^39^

Infected human gingival fibroblasts have been reported to exhibit various morphological alterations, such as extensive vacuolization and mitochondrial breakdown,^40^ and increased mtROS production was reportedly observed in macrophages infected with *P*. *gingivalis*.^32^ Additionally, Wu revealed increases in the cellular size, DNA fragmentation, nuclear condensation, and Bad, cytochrome c, and caspase-9 activities in cultured myocardial cells treated with *P*. *gingivalis*-conditioned medium.^41^ The study suggested that *P*. *gingivalis*-conditioned medium could activate mitochondrial-dependent apoptotic pathways, thereby leading to cell death in myocardial cells.

The results regarding the influence of *P*. *gingivalis* lipopolysaccharide (LPS) on mitochondria are not always consistent. For example, Li, Pedro Bullón, and Kiran Napa et al. believe that the treatment of fibroblast with *P*. *gingivalis* LPS leads to increased mtROS production, loss of MMP, reduced oxygen consumption, and mitochondrial biogenesis,^42–46^ and Zhu et al. obtained similar results in human gingival epithelial cells. The authors concluded that the ROS levels were increased significantly in cells treated with both high glucose and *P*. *gingivalis* LPS. Pretreatment with different concentrations of baicalein eliminated the production of ROS and the loss of MMP induced by high glucose in combination with LPS.^47^ Herath found that antioxidant proteins (such as mitochondrial manganese-containing superoxide dismutase and peroxiredoxin 5) were upregulated by LPS, and heterogeneous *P*. *gingivalis* LPS was shown to modulate the immunoinflammatory response, antioxidant defense, and cytoskeletal dynamics in human gingival fibroblasts.^48^

Based on all of the above viewpoints, it is reasonable to hypothesize that *P*. *gingivalis* differentially manipulates mitochondrial function depending on the cell type. Herein, we first clarified the detailed pathological effects of *P*. *gingivalis* on mitochondrial function in endothelial cells, revealing a series of features in the infected cells indicating mitochondrial dysfunction, including elevated mitochondrial fragmentation, increased mtROS levels, collapse of the MMP, and reduced ATP production. Furthermore, the underlying mechanism by which *P*. *gingivalis* infection promoted mitochondrial dysfunction was explored. We are the first to highlighted the potential implications of *P*. *gingivalis* in atherosclerosis progression in the context of mitochondrial function.

The mitochondrial fusion and fission machinery are critical for sustaining the function of mitochondria. As a main regulator of mitochondrial fission, the cytoplasmic protein Drp1 translocates to the outer mitochondrial membrane after activation.^25,49–51^ Because its recruitment to mitochondria is tightly regulated, Drp1 is considered an indicator of mitochondrial fission.^52^ Impaired or dysfunctional components of mitochondrial networks are isolated by mitochondrial fission or fragmentation. As stated by many researchers, p-Drp1 (Ser616) potentially mediates numerous diseases related to mitochondrial fragmentation. For instance, p-Drp1 (Ser616) was shown to promote transient global ischemia-induced neuronal damage, which was attenuated by Mdivi-1.^53^ Moreover, p-Drp1 (Ser616) levels were increased by nitric oxide, followed by the recruitment of p-Drp1 to mitochondria, thereby causing mitochondrial fragmentation.^54^ High glucose levels promoted ROCK1 activation and p-Drp1 (Ser616) expression, which are essential for mitochondrial fragmentation.^55^

Although the total Drp1 expression was not altered herein, its phosphorylation at Ser616 was increased. We also found that *P*. *gingivalis* promoted the translocation of Drp1 to mitochondria, accompanied by an increase in mitochondrial fragmentation. Moreover, Mdivi-1 successfully blocked mitochondrial fragmentation, mtROS accumulation, MMP loss, and ATP reduction caused by *P*. *gingivalis*. Thus, we proved that Drp1 is involved in regulating mitochondrial fission. To the best of our knowledge, we are the first to describe the role of Drp1 in mediating mitochondrial fission in endothelial cells with *P*. *gingivalis*-induced mitochondrial dysfunction. We should note that this study was solely focused on the influence of viable *P*. *gingivalis*, which simulates the conditions in the human body. It is well known that LPS, gingipains, fimbriae, capsules, and other virulence factors are among the strategies exploited by *P*. *gingivalis* to modulate a variety of host immune components or induce an inflammatory environment. The virulence factors responsible for mitochondrial dysfunction remain unknown. Our future work will focus on the effects of *P*. *gingivalis* LPS and gingipain activity on endothelial mitochondria.

We herein identified a novel role of *P*. *gingivalis* in manipulating mitochondrial shape and function via a mechanism that is dependent on Drp1. Taken together, the findings presented suggest that Drp1 potentially mediates the atherogenic response related to the pathogenesis of atherosclerosis induced by *P*. *gingivalis*.

## Materials and methods

### Cell culture

EA.hy926 cells (human umbilical vein endothelial cells) were purchased from Cellcook Biotech Company (Guangzhou, China) and cultured in Dulbecco’s modified Eagle’s medium (Gibco BRL, CA, USA) supplemented with 15% fetal bovine serum (GeneTimes, Shanghai, China). The cells were maintained at 37 °C in a 5% CO_2_ humidified incubator for use in subsequent assays.

### Bacterial strain and culture

*P*. *gingivalis* strain ATCC 33277 was cultured on brain heart infusion (BHI) broth agar plates containing 40% BHI, 10% agar, 0.5% hemin, 0.1% vitamin K_1_, and 20% defibrillated sheep blood under anaerobic conditions (80% N_2_, 10% O_2_, and 10% H_2_). EA.hy926 cells were infected with *P*. *gingivalis* at a multiplicity of infection (MOI) of 100 for a subsequent series of assays. Uninfected cells cultured under identical conditions were used as controls.

### Transmission electron microscopy

EA.hy926 cells (1 × 10^6^ cells per well) were seeded in six-well plates. After treatment, the cells were collected and fixed with 2.5% glutaraldehyde at 4 °C for 24 h, postfixed in 1% osmium tetroxide for 2 h, dehydrated in gradient ethanol, and then embedded in epoxy resin. Mitochondrial morphology was observed under a transmission electron microscope (H7650, Hitachi, Tokyo, Japan).

### Mitochondrial network quantification

EA.hy926 cells were cultured in a confocal Petri dish (1 × 10^5^ cells per dish). After treatment, the cells were washed and incubated with MitoTracker Red CMXRos (100 nmol·L^−1^, Solarbio, Beijing, China) for 0.5 h at 37 °C before being observed by CLSM (GeneTimes, Shanghai, China). The mitochondrial structures in the images were isolated processing, and the mitochondrial length, AR, and FF values were calculated by Image-Pro Plus 6.0. The AR depicts changes in mitochondrial length, whereas the FF depicts changes in the length and degree of branching. Both the FF and AR are independent of the image magnification.^56^ We used the “top-hat” filter to spatially process the graphics, removed the artifacts to construct binary graphics, and automatically extracted the mitochondrial parameters from the confocal graphics. The average mitochondrial length, AR, and FF values were calculated from three random images per experiment, and smaller values represented increased mitochondrial fragmentation.

### Measurement of mitochondrial mtROS production

MtROS levels were detected using MitoSOX Red (Yeasen, Shanghai, China), a mitochondrial superoxide indicator. The cells were washed and then stained with 5 µM MitoSOX Red in serum-free medium for 0.5 h. Afterward, the red fluorescence intensity was observed immediately by CLSM.

### Determination of the MMP

JC-1, a fluorescent probe (Bestbio, Shanghai, China), was used to detect the MMP. Briefly, the cells were incubated with JC-1 working buffer for 0.5 h, and images were obtained by CLSM. According to the manufacturer’s protocol, the probe can be used to detect two types of cellular fluorescence; healthy cells exhibit the red aggregate, and JC monomers appear as the MMP decreases, which eventually leads to a shift to green fluorescence. Representative pictures were captured by CLSM, and the ratio of red/green fluorescence intensity was used to estimate the MMP.

### Measurement of ATP production

ATP production was measured using a luciferase-based assay kit (Beyotime, Shanghai, China). Briefly, the cells were washed and lysed, followed by centrifugation at 12 000 × *g* at 4 °C for 5 min. The supernatants and ATP standard solutions were mixed with dilution buffer containing luciferase, and luminescence was detected using a microplate reader (Infinite M200, TECAN, Vienna, Austria). Then, the concentration of ATP was calculated according to the standard curve.

### Quantification of mtROS and MMP levels by flow cytometry

The mtROS and MMP levels were quantified by MitoSOX Red and JC-1 kits, respectively. The cells were resuspended in staining solution and stained as described above. Afterward, the cells were centrifuged and washed, followed immediately by flow cytometry analysis. The fluorescence intensity was quantified by a flow cytometer (FACS, Becton-Dickinson, Islandia, NY, USA) and FlowJo vX0.7 software.

### Western blot analysis of OPA1, Mfn1, Mfn2, Fis1, and Drp1

Cellular proteins were extracted and quantified using a BCA assay kit (Beyotime Biotech, Shanghai, China). Equal amounts of proteins were dissolved in SDS-PAGE buffer, separated by electrophoresis and transferred to polyvinylidene difluoride membranes. After blocking, the proteins were incubated with rabbit anti-OPA1 (1:800, ABclonal, MA, USA), rabbit anti-Mfn1 (1:1 500, ABclonal), rabbit anti-Mfn2 (1:1 500, ABclonal), rabbit anti-Fis1 (1:1 000, ABclonal), rabbit anti-Drp1 (1:1 000, ABclonal), and rabbit anti-p-Drp1 Ser616 (1:700, ABclonal) at 4 °C overnight, followed by incubation with a goat anti-rabbit IgG (1:100, Abbkine Inc., Redlands, CA, USA) secondary antibody. GAPDH (1:1 000, Affinity Biosciences, OH, USA) was used as the internal control. The results were analyzed using ImageJ 1.8.0 software.

### Inhibition of Drp1 with Mdivi-1

Mdivi-1 (Abcam, Cambridge, UK), a specific Drp1 inhibitor, was diluted in culture medium to a final concentration of 20 mmol·L^−1^ and added to the cells 2 h before *P*. *gingivalis* infection. The cells treated with only DMSO were used as negative controls. Finally, mitochondrial morphology and function were evaluated.

### Detection of mitochondria and p-Drp1 (Ser616) colocalization by immunofluorescence

EA.hy926 cells were grown on a confocal Petri dish (1 × 10^5^ cells per dish). After treatment, the cells were coincubated with MitoTracker Red CMXRos (200 nmol·L^−1^, red fluorescence for mitochondrial staining) at 37 °C for 0.5 h. After rinsing with PBS, the cells were fixed, permeated, blocked, and then incubated with rabbit anti-p-Drp1 Ser616 (1:400, CST, MA, US) overnight at 4 °C. The cells were then incubated with the secondary antibody goat anti-rabbit FITC (1:100, Solarbio, Beijing, China, green fluorescence for Drp1 staining) at room temperature for 1 h before being rinsed and immediately observed by CLSM.

### Statistical analysis

Statistical analysis was carried out using SPSS 17.0 software. All data are presented as the mean ± SD of three independent experiments. The means of two groups were compared by the pairwise Student’s *t*-test, while single-factor ANOVA and the least significant difference (LSD) test were applied to compare multiple groups. Post-hoc analysis was carried out using the Student-Newman-Keuls test. Differences with two-tailed probability (*P*) values <0.05 were considered significant.
