# Correction to: *Porphyromonas gingivalis* infection promotes mitochondrial dysfunction through Drp1-dependent mitochondrial fission in endothelial cells

**DOI:** 10.1038/s41368-021-00153-1

**Published:** 2022-01-12

**Authors:** Tong Xu, Qin Dong, Yuxiao Luo, Yanqing Liu, Liang Gao, Yaping Pan, Dongmei Zhang

**Affiliations:** 1 Department of Periodontics, School of Stomatology, China Medical University, Shenyang, China; 2 Department of Periodontics and Oral Biology, School and Hospital of Stomatology, China Medical University, Liaoning Provincial Key Laboratory of Oral Disease, Shenyang, China

**Keywords:** Mechanisms of disease, Bacterial pathogenesis

Correction to: *International Journal of Oral Science* 10.1038/s41368-021-00134-4, published online 03 September 2021

Following publication of this article^1^, it is reported this article contained the below two errors:

1. The caption of Fig. 3c should be corrected from “DAPDH” to “GAPDH”
2. In the sentence on the second last line on page 2, “after 6 h of infection” should be changed to “after 12 h of infection”.

The original article has been updated.
